# Genetic perspective on the synergistic connection between vesicular transport, lysosomal and mitochondrial pathways associated with Parkinson’s disease pathogenesis

**DOI:** 10.1186/s40478-020-00935-4

**Published:** 2020-05-06

**Authors:** Stefanie Smolders, Christine Van Broeckhoven

**Affiliations:** 1 Neurodegenerative Brain Diseases Group, VIB Center for Molecular Neurology, University of Antwerp – CDE, Universiteitsplein 1, 2610 Antwerpen, Belgium; 2 Biomedical Sciences, University of Antwerp, Antwerpen, Belgium

**Keywords:** Parkinson’s disease, Vesicular trafficking defects, Lysosomal dysfunction, Mitochondrial dysfunction, Lysosomal storage disorder genes, Oligogenic and polygenic inheritance

## Abstract

Parkinson’s disease (PD) and atypical parkinsonian syndromes (APS) are symptomatically characterized by parkinsonism, with the latter presenting additionally a distinctive range of atypical features. Although the majority of patients with PD and APS appear to be sporadic, genetic causes of several rare monogenic disease variants were identified. The knowledge acquired from these genetic factors indicated that defects in vesicular transport pathways, endo-lysosomal dysfunction, impaired autophagy-lysosomal protein and organelle degradation pathways, α-synuclein aggregation and mitochondrial dysfunction play key roles in PD pathogenesis. Moreover, membrane dynamics are increasingly recognized as a key player in the disease pathogenesis due lipid homeostasis alterations, associated with lysosomal dysfunction, caused by mutations in several PD and APS genes. The importance of lysosomal dysfunction and lipid homeostasis is strengthened by both genetic discoveries and clinical epidemiology of the association between parkinsonism and lysosomal storage disorders (LSDs), caused by the disruption of lysosomal biogenesis or function. A synergistic coordination between vesicular trafficking, lysosomal and mitochondria defects exist whereby mutations in PD and APS genes encoding proteins primarily involved one PD pathway are frequently associated with defects in other PD pathways as a secondary effect. Moreover, accumulating clinical and genetic observations suggest more complex inheritance patters of familial PD exist, including oligogenic and polygenic inheritance of genes in the same or interconnected PD pathways, further strengthening their synergistic connection.

Here, we provide a comprehensive overview of PD and APS genes with functions in vesicular transport, lysosomal and mitochondrial pathways, and highlight functional and genetic evidence of the synergistic connection between these PD associated pathways.

## Introduction

Approximately 1–2% of the worldwide population aged over 65 years is affected with PD, and up to 4–5% people aged over 85 years [71]. The mean age at onset is 70 years, although 5–10% of patients develop PD before the age of 50, referred to as early-onset PD (EOPD) [298]. Clinically, PD patients present with bradykinesia, muscle rigidity, resting tremor and gait instability [270]. Various non-motor symptoms may occur as well, including cognitive impairment and dementia, depression and apathy, excessive daytime sleepiness and insomnia, impulse control disorder, and autonomic dysfunctions [267]. PD symptoms manifests when approximately 30% of dopaminergic neurons in the *substantia nigra* have been degenerated [96, 120]. The most effective symptomatic treatment of PD consists of replenishing dopamine levels by administering the dopamine precursor levodopa though prolonged use could result in the development of adverse effects such as dyskinesias or wearing off [14]. Parkinsonian syndromes, of which PD is the most common one, are symptomatically defined by parkinsonism, comprising the four core motor symptoms of PD, in addition to a distinctive range of atypical features. Under this category are numerous heterogeneous syndromes that are often misdiagnosed as PD due to considerable overlap in symptoms especially early in the disease course [219].

Although the majority of patients with PD and APS appear to be sporadic, genetic causes of several rare monogenic disease variants were identified. The knowledge acquired from the protein products of identified causal genes and risk factors of PD and APS indicates that defects in vesicular transport pathways, endo-lysosomal dysfunction, impaired autophagy-lysosomal protein and organelle degradation pathways, α-synuclein aggregation and mitochondrial dysfunction play key roles in PD pathogenesis [2, 29, 121, 236, 371]. More recent advances have revealed that several parkinsonism associated genes regulate membrane dynamics wherein mutations cause lipid pathway alterations associated with lysosomal dysfunction [82, 93, 201, 213]. Additionally, associations between parkinsonism and lysosomal storage disorders (LSDs), caused by disruption of lysosomal biogenesis or function, are emerging from genetic discoveries and clinical epidemiology [82, 169].

This review focusses on these molecular pathways affected in PD and their increasingly recognized synergistic relationship in PD pathogenesis, which emerged from the identification of causal genes and risk factors contributing to the development of PD and related APS. Emerging observation suggest complex inheritance patterns of PD, including oligogenic and polygenic inheritance of gene variants of interconnected PD pathways, suggesting crosstalk between PD associated pathways.

### Vesicular transport pathways regulated by PD and APS genes

Intracellular vesicular transport pathways, which enables traffic of molecules between specific membrane-enclosed compartments, are especially vulnerable in neurons due to their highly complex organization of cell body and processes comprising axons, axon terminals and dendrites. Consequently, defect of vesicular transport pathways have been implicated in multiple neurodegenerative diseases [84, 354]. Several distinct pathways of complex, highly dynamic vesicular membrane structures with overlapping properties exist, including endocytosis, exocytosis, endosomal sorting and recycling, retrograde transport and autophagy. These membrane dynamics are important to maintain overall cellular homeostasis and organelle activities. During the regulation of membrane dynamics, the lipid and protein composition of the membranes changes. Several genes associated with PD and APS encode proteins that are involved in vesicular transport pathways. These genetic discoveries illuminate defects of the endosomal trafficking machinery and disrupted trafficking as pathological processes contributing to the development of PD.

#### LRRK2

**Fig. 1:**
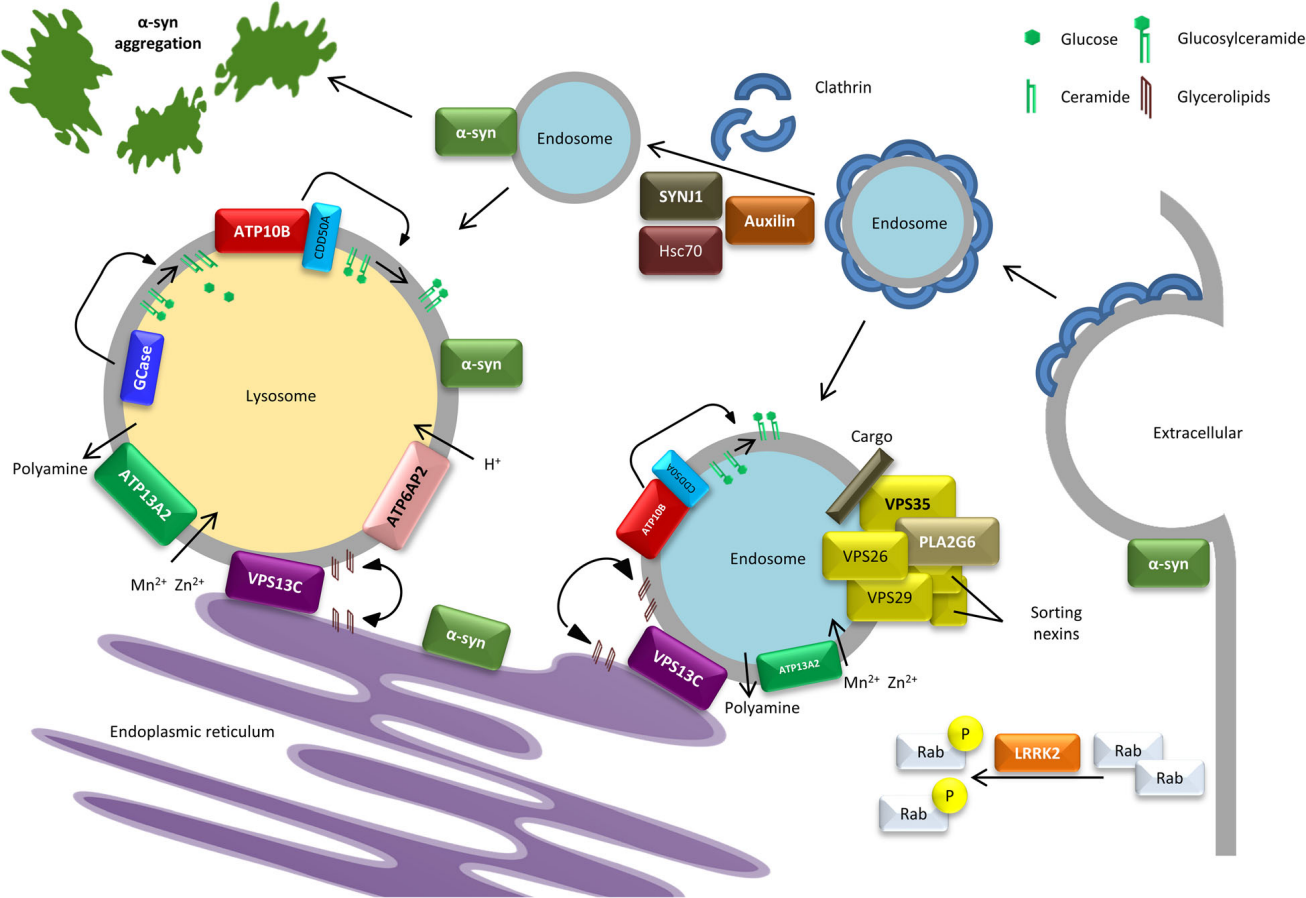
Schematic representation of vesicular transport and lysosomal pathways affected in Parkinson's disease. Mutations in α-synuclein (α-syn), LRRK2 and VPS35 are associated with autosomal dominant Parkinson's disease (PD), whereas mutations in VPS13C and ATP10B are associated with autosomal recessive PD. Mutations in ATP13A2, PLA2G6, DNAJC6, ATP6AP2 and SYNJ1 are associated with autosomal recessive atypical parkinsonian syndromes (APS). α-Synuclein interacts with membranes and functions in intracellular trafficking transport pathways. LRRK2 phosphorylates a subgroup of Rab GTPases which are important regulators of intracellular vesicle transport. VPS35, VPS26 and VPS29 form the retromer cargo-recognition complex involved in intracellular retrograde transport from endosomes to the trans-Golgi network, and associates with a dimer of sorting nexins. VPS13C tethers between the endoplasmic reticulum and late endosomes and lysosomes, and transports glycerolipids between membranes. ATP10B and ATP13A2 are both late endosomal/lysosomal P-ATPases, involved respectively in glucosylceramide export, and polyamine export/Mn^2+^ and Zn^2+^ import. ATP6AP2 is a subunit of the vacuolar H+ ATPase (V-ATPase) involved in maintaining a low lysosomal pH. PLA2G6 hydrolyzes the sn-2 ester bond of membrane glycerophospholipids to yield free fatty acids and lysophospholipids and interacts with the retromer subunits VPS35 and VPS26. DNAJC6 and SYNJ1 both play a crucial role in the detachment of the clathrin-coat after clathrin-mediated endocytosis

Autosomal dominant mutations in *LRRK2* are the most frequent cause of PD, accounting for 1–2% of all PD patients (Table 1) [251]. Although over 100 missense mutations in *LRRK2* have been reported, only a few are considered to be pathogenic based on co-segregation with the disease [251]. LRRK2 encodes a large, multi-domain protein containing a kinase, GTPase and protein-interaction domains. Recent research revealed that LRRK2 plays a role in vesicular transport, autophagy and lysosomal function [283]. LRRK2 is able to phosphorylate a subgroup of Rab GTPases, including Rab8A and Rab10 at highly conserved positions in the center of the effector-binding motif (Fig. 1) [325, 326]. Vesicle formation, vesicle motility along cytoskeleton elements, and docking and fusion at target membranes in the endocytic pathway is controlled by a complex regulatory machinery, which includes Rab GTPases in which Rab GTPases play a major role [25]. The pathogenic LRRK2 mutations cluster within the GTPase and kinase domains, resulting in an increased kinase activity [10]. Phosphorylation of Rab8A and Rab10 by LRRK2 prevents these Rab proteins to bind to downstream effectors, causing perturbations in vesicular transport due to pathogenic *LRRK2* mutations [325, 326]. Nevertheless, other regulatory mechanisms of vesicular transport may be affected as well by LRRK2 mutations. LRRK2 is localized in Rab5 positive early and Rab7 positive late endosomes, suggesting a role in endosomal trafficking as well as the autophagy lysosomal pathway [83, 117, 118, 140, 242].

**Table 1:** Genes implicated in Parkinson disease and atypical parkinsonian syndromes

Gene	MOI	Mutation spectrum	Mutation mechanism	Clinical phenotype	Levodopa response	Pathology	Protein product	Pathway	References
*ATP10B*	AR	Missense and splice site mutations	LOF	EOPD/LOPD	Limited, levodopa-induced dyskinesia	Unknown	Phospholipid transporting ATPase 10B	Endo-lysosome	[213]
*ATP13A2*	AR	Missense and PTC mutations	LOF	Juvenile APS called Kufor-Rakeb syndrome with pyramidal signs, supranuclear gaze palsy and cognitive impairment; NCL; HSP	Good	Lipofuscinosis	Cation transporting ATPase 13A2	Endo-lysosome	[37, 90, 257, 277]
*ATP6AP2*	XR	p.Ser115Ser and p.Asp107Asp	LOF	Juvenile APS with slow disease progression and considerable phenotypic variability including spasticity, intellectual disability and epilepsy	Limited, levodopa-induced dyskinesia	LB-, tau+^b^	Renin/prorenin receptor	Endo-lysosome	[176, 279]
*DJ-1*	AR	Deletions, PTC and missense mutations	LOF	EOPD with slow disease progression and rarely autonomic dysfunctions or cognitive impairment	Limited, levodopa-induced dyskinesia	LB + ^b^	DJ-1	Mitochondria	[3, 31, 163, 334].
*DNAJC6*	AR	c.802-2A > G, p.Thr741Thr, p.Gln791^b^, p.Gln846^b^, p.Arg927Gly	LOF	Juvenile and early-onset APS with rapidly disease progression and possible intellectual disability, seizures and pyramidal signs.	Limited, levodopa-induced dyskinesia and psychiatric features	Unknown	Auxilin	Vesicular transport	[85, 175, 240]
*FBXO7*	AR	PTC and missense mutations	LOF	Ranging from classic EOPD to juvenile APS with pyramidal signs (spasticity, impaired fine movements and increased reflexes)	Limited, levodopa-induced dyskinesia and psychiatric features	Unknown	F-box protein 7	Mitochondria	[76, 125, 149, 204, 311, 369]
*PARK2*	AR	Deletions, PTC and missense mutations	LOF	EOPD with slow disease progression and rarely autonomic dysfunctions or cognitive impairment	Limited, levodopa-induced dyskinesia	Most LB-	Parkin	Mitochondria	[1, 156, 160, 264]
*PINK1*	AR	Deletions, PTC and missense mutations	LOF	EOPD with slow disease progression and rarely autonomic dysfunctions or cognitive impairment	Limited, levodopa-induced dyskinesia	LB + ^b^	PTEN-induced kinase 1	Mitochondria	[156, 293]
*PLA2G6*	AR	CNV, PTC and missense mutations	LOF	Early-onset APS called dystonia-parkinsonism with cognitive decline, autonomic dysfunction and psychiatric manifestations; INAD; atypical NAD	Limited, levodopa-induced dyskinesia	Axonal spheroid, iron deposits	Phospholipase A2	Endo-lysosome	[126, 139, 157, 161, 184, 249, 316, 370]
*LRRK2*	AD	Missense mutations	GOF	LOPD with slow disease progression and rarely cognitive impairment	Good	Most LB+, rarely tau+	Leucine-rich repeat kinase 2	Vesicular transport	[132, 251, 285, 346, 379]
*SNCA*	AD	Multiplications, p.Ala30Pro, p.Glu46Lys, p.Gly51Asp, p.Ala53Glu and p.Ala53Thr^a^	GOF	EOPD/LOPD with severe, rapidly disease progression and cognitive impairment; DLB; MSA	Good	LB+	α-Synuclein	Vesicular transport	[6, 28, 162, 181, 191, 263, 272, 284, 295, 317, 372]
*SYNJ1*	AR	p.Arg258Gln, p.Arg459Pro	LOF	Juvenile APS with possible cognitive impairment, epilepsy and dystonia	Limited, levodopa-induced dyskinesia	LB-, tau+^b^	Synaptojanin 1	Vesicular transport	[168, 180, 239, 273]
*VPS13C*	AR	Deletions and PTC mutations	LOF	EOPD/DLB with severe, rapidly disease progression and cognitive decline	Good	LB+	Vacuolar protein sorting 13C	Endo-lysosome	[69, 183, 193, 297]
*VPS35*	AD	p.Asp620Asn	LOF	LOPD with slow disease progression and rarely cognitive impairment or neuropsychiatric symptoms	Good	Unknown	Vacuolar protein sorting 35	Vesicular transport	[275, 364]

^a^Pathogenicity of the *SNCA* p.His50Gln is uncertain [28]. ^b^Neuropathological report of a single carrier. Abbreviations: MOI, mode of inheritance; *AR* autosomal recessive, *AD* autosomal dominant, *XR* X-linked recessive, *EOPD* early-onset Parkinson disease; LOPD, late-onset Parkinson disease, *APS* atypical parkinsonian syndrome; *MSA* multiple system atrophy, *DLB* dementia with Lewy bodies; *NCL* neuronal ceroid-lipofuscinosis, *HSP* hereditary spastic paraplegia, *INAD* infantile neuroaxonal dystrophy, *NAD* neuroaxonal dystrophy, *PTC* premature termination codon, *CNV* copy number variation, *LB+* positive for Lewy body pathology, *LB*- negative for Lewy body pathology; tau+, positive for tau pathology

#### VPS35

The *VPS35* p.Asp620Asn mutation is a rare cause of autosomal dominant inherited PD, with a prevalence of around 0.115% (Table 1) [275]. While other variants in *VPS35* have been identified as well, the p.Asp620Asn mutation is the only recurrent mutation segregating with PD in different populations [275, 364]. *VPS35* encodes a core component of the retromer cargo-recognition complex (CRC) involved in intracellular retrograde transport from endosomes to the trans-Golgi network [103]. The CRC trimer consists of VPS26, VPS29 and VPS35, and associates with a dimer of sorting nexins which is facilitated by Rab7 (Fig. 1) [177, 208, 301, 302]. In addition to carrying cargo from endosomes to the trans-Golgi network, the retromer carries cargo from endosomes to plasma membranes to recycle membrane bound receptors [343]. The cation-independent mannose 6-phosphate receptor (CI-MPR) is a carrier protein of the retromer system, involved in trafficking lysosomal proteases, such as the cathepsin D (CTSD), to lysosomes [40, 300]. In the trans-Golgi network, the mannose-6-phosphate residues of the CTSD signal peptide are recognized by CI-MPR and initiate trafficking towards the endosome [222]. Inside the endosome, CTSD is activated by proteolytic cleavage of the signal peptide and released for transport to the lysosome. Subsequently, CI-MPR is recycled from the endosome to the trans-Golgi network [187, 222]. The dominant negative VPS35 p.Asp620Asn mutation causes retromer dysfunction and a decreased delivery of CTSD to the lysosome, contributing to lysosomal dysfunction [98, 103]. Moreover, α-synuclein is known to be transported by the retromer complex, and knockdown of VPS35 in *Drosophila* leads to the accumulation of α-synuclein within the neurons [222].

#### DNAJC6

Missense and premature termination codon (PTC) mutations in DNAJC6, in line with autosomal recessive inheritance, have been reported in juvenile and early-onset APS called dystonia-parkinsonism (Table 1) [85, 87, 175, 240]. *DNAJC6* encodes the neuron specific isoform of the co-chaperone auxilin-1, which plays a crucial role in the detachment of the clathrin-coat after clathrin-mediated endocytosis (Fig. 1). Auxilin-1 consists of a phosphoinositide phosphatase PTEN-like domain, which is required for the recruitment to a clathrin-coated pit, a clathrin-binding domain, and a J domain, which enables its interaction with Hsc70, a chaperone involved in diverse cellular processes [188]. The c.801-2A > G mutation in *DNAJC6* generates two abnormal transcripts that lack either a significant part of the J domain or the PTEN-like domain, the p.Gln734* deletes 180 amino acid residues at the C-terminus of the protein, and the pathogenic p.Arg927Gly missense mutation in the J domain is predicted to reduce the positive charge on the protein surface, suggesting a loss-of-function mechanism for all pathogenic *DNAJC6* mutations [85, 175, 240].

#### SYNJ1

The homozygous *SYNJ1* p.Arg258Gln and p.Arg459Pro were identified in families affected with autosomal recessive juvenile APS (Table 1) [168, 180, 239, 273]. *SYNJ1* encodes synaptojanin 1, a phosphoinositide phosphatase with a major role in endocytic recycling of synaptic vesicles [273]. SYNJ1 contains two different phosphatase domains, the 5-phosphatase and the Sac1 domain, which target different phosphoinositide phosphates (PIPs), and cooperates with Auxilin and Hsc70 to remove the clathrin-coat after clathrin-mediated endocytosis (Fig. 1) [180, 273]. The 5-phosphatase domain regulates synaptic vesicle endocytosis by dephosphorylating phosphatidylinositol 4,5-bisphosphate (PIP_2_) to facilitate uncoating of clathrin coated vesicles [60, 211]. Knock-in of the *SYNJ1* p.Arg258Gln mutation in mice leads to accumulation of auxilin, clathrin and parkin, and impaired synaptic vesicle endocytosis in neurons [46].

### PD and APS genes with endo-lysosomal functions

Lysosomes are the endpoint of various degradation pathways including endocytosis and autophagy and contain nearly 60 different hydrolytic enzymes including nucleases, proteases, phosphatases, lipases, sulfatases amongst others to degrade macromolecules and cellular components [290]. Many factors regulate lysosomal function including an acidic internal pH at which lysosomal hydrolases are active through the activity of vacuolar H+ ATPase (V-ATPase) [290]. Neurons are especially vulnerable to lysosomal dysfunction because, without the aid of cell division, they are largely dependent on autophagy to prevent the accumulation of cellular protein and damaged organelles. Insufficient degradation of neurotoxic proteins by lysosomes has been implicated in multiple neurodegenerative diseases [30]. Two novel PD genes, *VPS13C* and *ATP10B*, and several APS genes encode proteins involved in endo-lysosomal functions.

#### VPS13C

The vacuolar protein sorting 13 C (*VPS13C*) gene was first identified as a new risk gene for PD in a meta-analysis of genome-wide association studies (GWAS) [230], and later homozygous and compound heterozygous PTC mutations in *VPS13C* were associated with a distinct form of early-onset parkinsonism characterized by rapid and severe disease progression and early cognitive decline [193]. Two independent studies confirmed VPS13C loss-of-function mutations in autosomal recessive PD [69, 297]. To date, the mutation spectrum includes PTC mutations, and a large deletion comprising multiple exons (Table 1) [69, 193, 297]. The human VPS13 family consists of four proteins, VPS13A/Chorein, VPS13B, VPS13C and VPS13D, with all family members having a strong homology to yeast Vps13. Yeast studies have suggested that Vps13 may have a role in lipid exchange between organelles and showed that yeast mutants lacking Vps13 causes defects in mitochondrial membrane integrity [186, 259]. More recent research showed that human VPS13C is a lipid transport protein that functions as a tether between the endoplasmic reticulum (ER) and late endosomes and lysosomes, and between the ER and lipid droplets, enabling transport of glycerolipids between membranes (Fig. 1) [183]. Subsequently, loss of VPS13C implicates defects in membrane lipid homeostasis and lysosomal dysfunction. Interestingly, loss-of-function mutations in other human VPS13 genes are associated with different recessive neurological disorders [67, 174, 304].

#### ATP10B

We recently identified compound heterozygous loss-of-function mutations in the ATPase class V type 10B (*ATP10B*) gene increasing risk for PD (Table 1) [213]. *ATP10B* mRNA is mainly expressed in the gastrointestinal track and the brain [213]. Approximately 65% PD patients develop gastrointestinal disorders 4 years after diagnosis and Lewy body pathology is also observed in the enteric nervous system of PD patients [35, 210, 353]. Previously, ATP10B was identified as a P4-type transport ATPase present in the late endo−/lysosomal compartment [228]. P4 ATPases are lipid flippases that use ATP to drive the transport of lipids from the lumen to the cytosolic membrane leaflet, establishing the vitally important lipid asymmetry between two membrane leaflets [11, 254]. ATP10B forms a heteromeric complex with the Cell Cycle Control Protein 50A (CDC50A) to facilitate the trafficking from the ER to the late endosome and lysosome [11]. We established that *ATP10B* is involved in the translocation of glucosylceramide (GluCer) and phosphatidylcholine (PC) towards the cytosolic membrane leaflet of late endosomes/lysosomes (Fig. 1) [213]. Moreover, ATP10B might also transport glucosylsphingosine and sphingomyelin besides GluCer and PC [213]. ATP10B functionally belongs to the ATP10A/D sub-class of human lipid flippase isoforms that share highly conserved functional domains for GluCer and PC transport, pointing to a physiological role of the ATP10A, B and D transporters in GluCer/PC uptake or subcellular redistribution [281]. However, so far no neurological diseases are associated with ATP10A or ATP10D. In cellular overexpression models, the identified PD associated ATP10B mutants were shown to be catalytically inactive and failed to provide cellular protection against the environmental PD risk factors rotenone and manganese [213]. In isolated cortical neurons, knockdown of ATP10B led to a significant loss of lysosomal mass and a higher lysosomal pH resulting in a global reduction of lysosomal degradative capacity. Rotenone exposure in ATP10B knockdown cortical neurons also impaired lysosomal membrane integrity, which is a major driver of lysosome dependent cell death [5].

#### ATP13A2

Loss-of-function mutations in *ATP13A2* cause Kufor-Rakeb syndrome, a rare form of juvenile onset autosomal recessive APS (Table 1) [277]. Pathogenic mutations in *ATP13A2* are as well identified in patients with neuronal ceroid lipofuscinoses, a neurodegenerative LSD, and patients with hereditary spastic paraplegia [37, 90]. The considerable clinical heterogeneity of *ATP13A2* mutation carriers could be partially explained by variable impact of different mutations on protein expression and functionality of ATP13A2 [257]. Interestingly, ATP13A2 has been identified in Lewy bodies in brains of sporadic PD patients [72, 226].

*ATP13A2* encodes a P-type ATPase, mainly localized at endosomes and lysosomes, with a role in manganese (Mn^2+^) and zinc (Zn^2+^) homeostasis, mitochondrial bioenergetics, and the autophagy lysosomal pathway (Fig. 1) [23, 128, 258, 336, 345]. Recently, ATP13A2 was identified as a lysosomal polyamine exporter with a high affinity for spermine [348]. The protein is highly expressed in the brain, especially in the *substantia nigra*. Most of the pathogenic missense mutations occur in functional domains of ATP13A2, including the transmembrane domains and the E1-E2 ATPase domain, resulting in a loss of protein function [257]. Patient derived cells of *ATP13A2* mutation carriers revealed an impaired Zn^2+^ homeostasis, with lysosomal and mitochondrial dysfunction as a consequence [258, 345]. Expression of wild-type but not mutant ATP13A2 protects mammalian cell lines and primary rat neuronal cultures against manganese induced cell death, also known as a PD environmental risk factor [336]. High concentrations of polyamines was shown to induce cell toxicity, which exacerbated by ATP13A2 loss due to lysosomal dysfunction [348]. Additionally, ATP13A2 has been shown to be involved in α-synuclein metabolism [205] and lipid homeostasis [212].

#### PLA2G6

Autosomal recessive mutations in the phospholipase A2 group 6 gene (*PLA2G6*) are causative for phospholipase A2-associated neurodegeneration (PLAN) syndromes, including classic infantile neuroaxonal dystrophy (INAD) and atypical neuroaxonal dystrophy with childhood-onset (atypical NAD), and adult onset APS called dystonia-parkinsonism, which is associated with Lewy bodies and neuroaxonal dystrophy (Table 1) [139, 157, 161, 249, 316, 370].

Mutations responsible for loss of *PLA2G6* catalytic activity usually lead to INAD and atypical NAD whereas mutations altering substrate preference or regulatory mechanisms are usually causal for adult onset dystonia-parkinsonism [88]. However, patients carrying the same *PLA2G6* mutation with different clinical phenotypes have been reported [225, 316]. The protein encoded by *PLA2G6* is a calcium-independent group VI phospholipase A2 (iPLA-β), which hydrolyzes the sn-2 ester bond of membrane glycerophospholipids to yield free fatty acids and lysophospholipids (Fig. 1) [17]. iPLA-β expression is enriched in dendrites and axon terminals [241]. PLA2G6 is involved in repair of oxidative damage to membrane phosphopholipids, membrane remodeling and iron homeostasis [17, 309]. Recently, Lin and colleagues demonstrated that the fly homolog of iPLA-β binds the retromer subunits VPS35 and VPS26, and that loss of iPLA-β impairs retromer function, causes lysosomal ceramide accumulation, and leads to lysosomal dysfunction [200].

#### ATP6AP2

Two synonymous variants in *ATP6AP2,* p.Ser115Ser and p.Asp107Asp, increasing exon 4 skipping, were identified in patients with X-linked APS characterized by parkinsonism, spasticity, intellectual disability and epilepsy (Table 1) [176, 279]. *ATP6AP2* encodes the lysosomal renin/prorenin receptor, an accessory unit of vacuolar H^+^ ATPase (V-ATPase) required for lysosomal degradative functions and autophagy (Fig. 1). Alterations in *ATP6AP2* are involved in different human phenotypes, suggesting a critical function in various organ systems [44, 127, 135, 144, 176, 245, 279, 287, 358]. ATP6AP2 interacts with renin/prorenin at the cell membrane which enhances proteolytic activity toward Angiotensin II and causes activation of intracellular signaling pathways resulting in secretion of inflammatory and fibrotic factors [274]. Consistent with its role in renin signaling, *ATP6AP2* polymorphisms have been linked to hypertension [135, 245, 358]. Moreover, as an accessory unit of the membrane transporter H^+^ ATPase, ATP6AP2 is involved in maintaining a low lysosomal pH and, thereby, degradation of cellular waste [255]. siRNA knockdown of *ATP6AP2* in HEK293 cells results in perturbed autophagy, inhibited lysosomal clearance and in the accumulation of autophagosomes, suggesting that the impaired autophagy in *ATP6AP2* mutation carriers is due to reduced vacuolar H^+^ ATPase activity [176]. Moreover, ATP6AP2 is a component of the WNT receptor complex involved in the canonical WNT signal transduction pathway in *Drosophila* and *Xenopus* [39, 63].

### α-Synuclein aggregation connected to defects in vesicular transport and autophagy-lysosomal dysfunction

The autophagy-lysosomal pathway is one of the two major degradation pathways present in the cell for identifying and delivering cytosolic components to the lysosome for degradation and recycling [79]. Macro-autophagy is involved in the degradation of aggregated proteins and damaged organelles, via engulfment by a phagophore membrane, subsequent maturation into a vesicle called the autophagosome and afterwards fusion with a lysosome. Meanwhile, micro-autophagy is characterized by direct lysosomal engulfment of cytosolic material into the lysosomes via the formation of invaginations of the lysosomal membrane. Chaperone-mediated autophagy is involved in the degradation of soluble monomeric proteins containing the penta-peptide motif KFERQ, via transport to the lysosome by the chaperone HSC70.

PD is pathologically characterized by the presence of Lewy bodies and Lewy neurites, composed mainly of amyloid fibrils of α-synuclein, in neurons [78] of the central nervous system, e.g. basal ganglia, the dorsal motor nucleus of the vagus, the olfactory bulb, the locus coeruleus, and of the peripheral nervous system, e.g. the enteric nervous system [35, 353]. α-Synuclein, encoded by *SNCA*, was initially linked to PD as the main component of Lewy bodies [323] and subsequently, dominant mutations in *SNCA* were identified as the first genetic cause of familial PD (Table 1) [268]. Pathogenic *SNCA* mutations are present in approximately 1–2% familial and 0.2% sporadic PD patients [95, 192, 238]. *SNCA* mutations are also implicated in dementia with Lewy bodies (DLB) [124, 243], and single nucleotide polymorphisms (SNPs) in *SNCA* are associated with multiple system atrophy, an APS pathologically characterized by the presence of α-synuclein immunoreactive glial cytoplasmic inclusions [6, 263, 295].

α-Synuclein is abundantly expressed in the central nervous system, with a prominent presynaptic localization, as a lipid binding protein that interacts with membranes (Fig. 1) [70, 143]. The function of α-synuclein remains poorly understood but involves maturation of pre-synaptic vesicles, synaptic vesicle recycling, regulation of neurotransmitter release, and plasticity of dopaminergic neurons [202, 206, 233]. The protein contains an N-terminal domain, which includes an imperfect conserved repeat KTKGEV and acts as the membrane anchor region, and a central non-amyloid β component domain consisting of hydrophobic residues that renders α-synuclein susceptible to polymerization [115]. The latter domain also behaves as a lipid sensor and determines the membrane binding affinity of α-synuclein [102]. Under normal physiological conditions α-synuclein occurs as a monomeric disordered protein which could shift upon membrane binding to an amphipathic helical structure [347]. Nevertheless, α-synuclein can also convert from the disordered, monomeric form to polymerized β-sheets constructed of additional recruited α-synuclein monomers, which will eventually lead to the formation of protofilaments and amyloid fibrils (Fig. 1) [59]. α-Synuclein can be degraded by both chaperone-mediated autophagy and micro-autophagy, with both pathways reported to be impaired in PD pathogenesis, resulting in α-synuclein accumulation [64]. Meanwhile, studies in both animal models and human induced pluripotent stem cell (iPSC) derived dopaminergic neurons have shown that elevated levels of α-synuclein disrupt numerous intracellular trafficking transport pathways, including at the ER, early and late endosomes, and lysosomes [58, 216, 247]. α-Synuclein expression in yeast resulted in an early block in ER to Golgi vesicular trafficking [58, 247]. In human midbrain synucleinopathy models, generated through lentiviral overexpression of α-synuclein in control cultures, or through the generation of patient lines harboring PD-causing mutations, α-synuclein accumulation was found to reduce lysosomal degradation capacity by disrupting trafficking of lysosomal hydrolases [216]. Moreover, α-synuclein accumulation disrupted the ER-Golgi localization of Rab1a, a key mediator of vesicular transport [216].

### PD and APS genes involved in mitochondrial pathways and mitophagy

Mitochondria are essential energy producing organelles that regulate cellular energy homeostasis and cell death. Mitochondria are highly dynamic and undergo fission and fusion to maintain a functional mitochondrial network [221]. Mitophagy, a process involved in the selective removal of damaged mitochondria through macro-autophagy, is therefore crucial for maintaining proper cellular functions. Mitophagy comprises three important steps: the recognition of impaired mitochondria and the formation of autophagic membranes, the engulfment by a phagophore membrane and subsequent maturation into a mitoautophagosome, and the fusion of the mitoautophagosome with a lysosome [51]. Defects in the autophagy-lysosomal pathway consequently lead to inappropriate removal of damaged mitochondria.

**Fig. 2:**
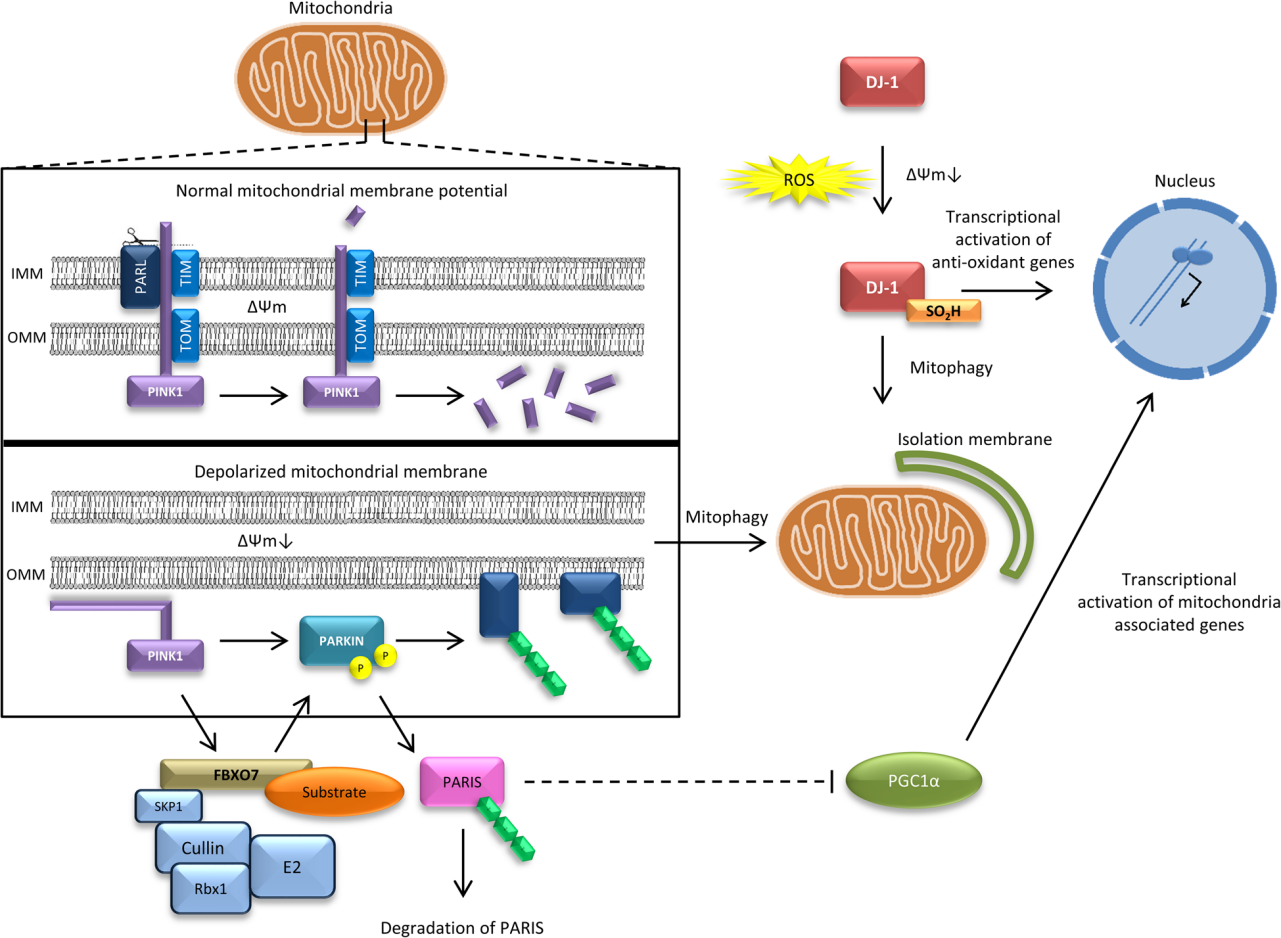
Schematic representation of the mitochondrial and oxidative stress pathways affected in Parkinson disease. Mutations in parkin, PINK1, DJ-1 and FBXO7 have been associated with autosomal recessive Parkinsonian syndromes. **a** In healthy, polarized mitochondria (∆Ψm) PINK1 translocates at the inner mitochondrial membrane via the mitochondrial import receptor TOMM20 machinery, which subsequently results in the degradation of PINK1. In damaged, depolarized mitochondria (∆Ψm↓), PINK1 accumulates at the outer mitochondrial membrane and recruits parkin upon phosphorylation. Moreover, parkin mediates the degradation of the parkin interacting substrate (PARIS), a repressor of the PGC1α transcriptional coactivator, leading to nuclear translocation of PGC1α and transcriptional activation of mitochondria associated genes. **b** In oxidative stress conditions or ∆Ψm↓, DJ-1 p.Cys106 will form a sulfonic acid, which will activate DJ-1 to regulate transcription of antioxidant genes and to promote mitophagy. **c** FBXO7 is a subunit of the SKP1-cullin-F-box (SCF) complex. PINK1 is involved in the recruitment of FBXO7 to damaged mitochondria which in turn leads to the recruitment of parkin

Mitochondrial dysfunction is known to contribute to several neurodegenerative diseases, including PD [121, 148]. A reduction in complex I mitochondrial respiratory chain activity was observed in in vivo and in vitro models of PD as well as in post-mortem brain tissue of idiopathic PD patients implicating a role for mitochondrial dysfunction in PD pathogenesis [256, 261, 294]. Later, loss-of-function homozygous and compound heterozygous mutations in *PARK2*, *PINK1*, *PARK7*/*DJ*-1 were found to be responsible for autosomal recessive EOPD. The proteins encoded by *PARK2* (parkin), *PINK1* and *DJ*-*1* have various well described functions but appear to converge towards mitochondrial function, including mitophagy, mitochondrial dynamics and oxidative stress control. Moreover, *FBXO7*, in which autosomal recessive mutations cause juvenile Parkinsonian-pyramidal syndrome, is connected to the *PARK2*/*PINK1* mitochondrial pathway (Fig. 2, Table 1).

#### PARK2

Bi-allelic mutations in *PARK2* are the most common cause of autosomal recessive PD (Table 1) [264]. Over 120 loss-of-function mutations have been identified so far, explaining approximately 15% of EOPD patients with an age at onset of 40–50 years [1]. Parkin, encoded by *PARK2*, plays a central role as a cytosolic E3 ubiquitin-ligating enzyme, and works in together with E1 ubiquitin-activating and E2 ubiquitin-conjugating enzymes of the ubiquitin proteasome system to ubiquitinate misfolded, damaged or unwanted proteins for degradation [307]. Later it was discovered that parkin is selectively recruited to impaired mitochondria during stress or upon membrane depolarization (Fig. 2a) [231]. Once localized at the outer mitochondrial membrane of depolarized mitochondria, parkin promotes the ubiquitination of outer mitochondrial membrane proteins involved in the upregulation of mitochondrial fusion, mitofusin 1 and mitofusin 2 (Fig. 2a) [112, 269]. The subsequent removal of these proteins would shift mitochondrial dynamics of fission and fusion and will eventually lead to mitochondrial fragmentation. Fragmented mitochondria are subsequently removed via mitophagy [48, 189]. Additionally, parkin regulates mitochondrial biogenesis by mediating the degradation of the parkin interacting substrate (PARIS), a repressor of the peroxisome proliferator-activated receptor gamma coactivator 1-α (PGC1α) transcriptional coactivator, leading to nuclear translocation of PGC1α and transcriptional activation of mitochondria associated genes [308, 328]. Interestingly, loss of dopaminergic neurons have been demonstrated in animal models in which the PGC1α gene was silenced or knocked out [146, 235].

#### PINK1

Mutations in the PTEN-induced putative kinase 1 gene (*PINK1*) are the second leading causes of recessive PD (Table 1). The prevalence of homozygous and compound heterozygous *PINK1* mutations varies from 0 to 4% [32, 54, 101, 170, 197, 238, 335, 361]. PINK1 is a highly conserved putative serine/threonine protein kinase localized to mitochondria via its mitochondrial targeting sequence at the N-terminus, and recognizes mitochondrial depolarisation, reactive oxygen species (ROS), or protein misfolding [107, 310, 355]. Most pathogenic mutations in *PINK1* are found in the serine/threonine kinase domain, suggesting loss of kinase activity plays a crucial role in the pathogenesis of *PINK1*-associated PD. In healthy mitochondria, the electrical polarization of the inner mitochondrial membrane ensures PINK1 to translocate at the inner mitochondrial membrane via the mitochondrial import receptor TOMM20 machinery. Here, PINK1 is rapidly cleaved by mitochondrial proteases, including the rhomboid protease presenilin-associated rhomboid-like protein (PARL), and subsequently degraded by the ubiquitin-proteasome system in the cytosol (Fig. 2a) [130]. In damaged, depolarized mitochondria, translocation of PINK1 by the TOMM20 machinery is inhibited, resulting in the accumulation of PINK1 at the outer mitochondrial membrane. Uncleaved PINK1 recruits and activates parkin by phosphorylating both ubiquitin and the ubiquitin-like domain of parkin (Fig. 2a) [153, 158, 178]. Loss-of-function of either parkin or PINK1 results in the accumulation of dysfunctional mitochondria in the cytoplasm, resulting in oxidative stress and subsequently cell death [91, 110, 113, 119, 253].

#### DJ-1

Approximately 0.4–1% of EOPD is caused by homozygous or compound heterozygous loss-of-function mutations in *DJ-1* (Table 1) [31, 163]. The protein encoded by *DJ-1* is involved in transcriptional regulation, oxidative stress responses, anti-apoptotic signaling and protein quality control within the neuronal cells [13, 43, 52, 114, 246, 352]. In addition, DJ-1 is required for the degradation of dysfunctional mitochondria via mitophagy [357]. DJ-1 is predominantly located in the cytoplasm and to a lesser extent at mitochondria and in the nucleus [151, 376]. However, in oxidative stress conditions, the translocation of DJ-1 into the nucleus is enhanced. Simultaneously, the highly conserved cysteine residue (p.Cys106) of DJ-1 will form a sulfonic acid (SOH, SO_2_H), which will activate DJ-1 to regulate transcription of antioxidant genes (Fig. 2b) [13, 43, 166, 167]. Additionally, upon oxidative stress and a decrease in mitochondrial membrane potential, DJ-1 associates with mitochondria promoting mitophagy through mechanisms that are still unknown (Fig. 2b) [152, 357]. Knockdown of DJ-1 results in increased ROS, decreased mitochondrial membrane potential and changes in mitochondrial morphology, leading to cell death [12, 26, 179].

#### The parkin/PINK1/DJ-1 mitochondrial pathway

The functions of parkin, PINK1 and DJ-1 intersect at the mitochondria, but whether DJ-1 is directly or indirectly implicated in a common pathway involving parkin and PINK1 is still inconclusive. For example, DJ-1 is not necessary for the recruitment of parkin to depolarized mitochondria and is not able to rescue the mitochondrial defects caused by loss of parkin [131, 218, 341]. However, DJ-1 is able to reduce damage from the mitochondrial complex I inhibitor rotenone in the absence of PINK1, without altering PINK1 mitochondrial phenotypes [341]. Meanwhile, both PINK1 and parkin can rescue the mitochondrial fragmentation caused by the loss of DJ-1 in primary neurons and immortalized cells [49, 142, 341]. These data suggest that DJ-1 acts in parallel to the PINK1/parkin pathway to control mitochondrial function and mitophagy.

#### FBXO7

Loss-of-function mutations in the F-box protein 7 gene (*FBXO7*) are responsible for autosomal recessive APS with various heterogenic phenotypes (Table 1) [76, 311]. FBXO7 is a subunit of the SKP1-cullin-F-box (SCF) complex that acts as a multimeric E3 ubiquitin ligase, wherein cullin1 and SKP1 constitute the core of the E3 ligase and FBXO7 functions as substrate-recruiting subunit. To ubiquitinate a substrate in the ubiquitin proteasome pathway, the SCF complex brings the E2-ubiquitin-conjugate and the substrate in close proximity [149]. Interestingly, FBXO7 contains at its N-terminus an ubiquitin-related (UbR) domain, which mediates the interaction with parkin to regulate mitochondrial quality control [42]. Indeed, mediated by PINK1, FBXO7 is translocated to damaged mitochondria where it is required for a successful recruitment of parkin (Fig. 2) [42]. *Drosophila* models showed that overexpressing FBXO7 suppresses mitochondrial disruption and neurodegeneration in *PARK2* mutants, confirming that they share a common role in mitochondrial biology [42, 378].

### Lysosomal dysfunction and lipid homeostasis alterations in PD: insights from lysosomal storage disorders genes

**Fig. 3:**
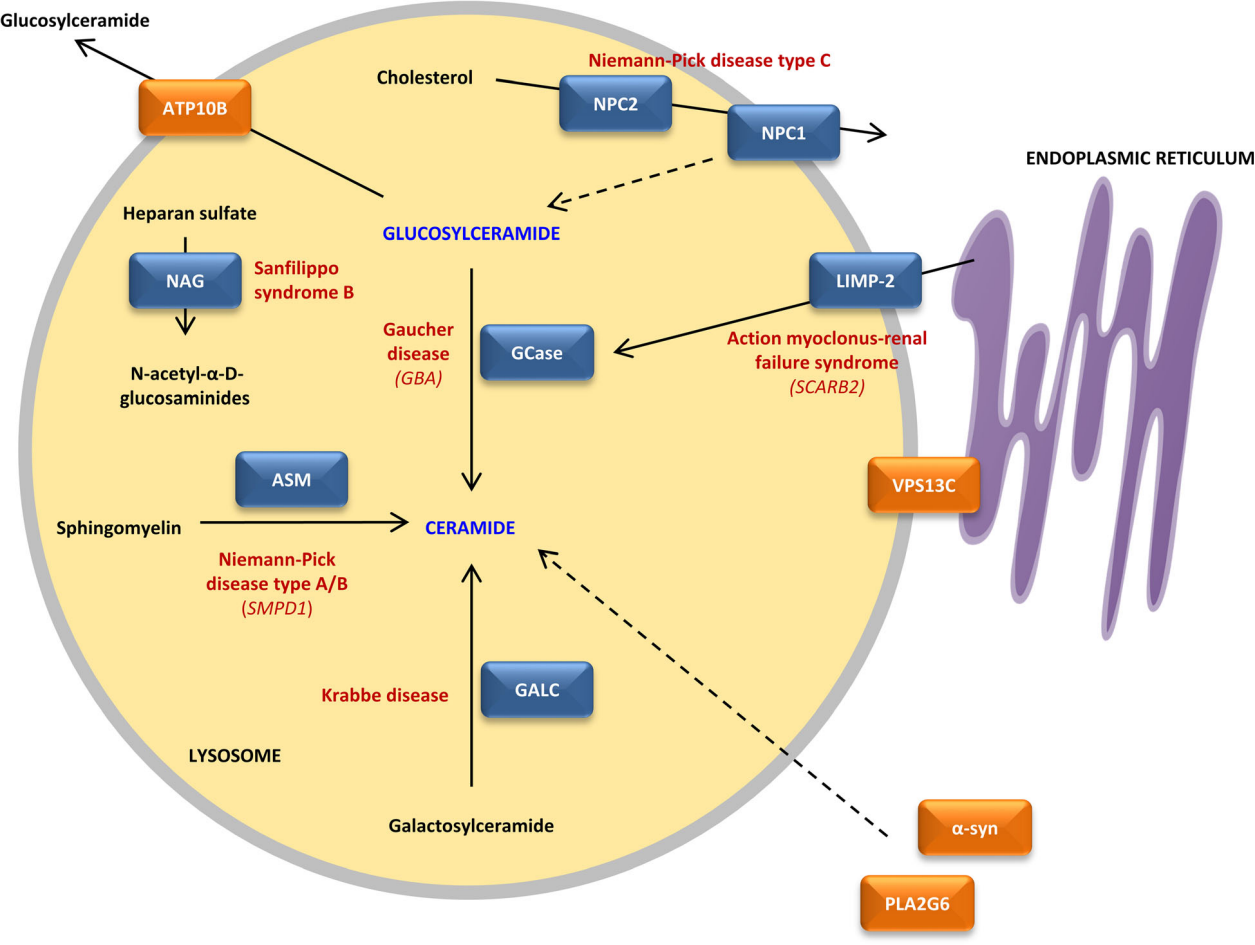
Lipid metabolism and lysosomal storage disorders associated with Parkinson's disease. Proteins indicated in orange are associated with Parkinson's disease (PD) and/or atypical Parkinsonian syndromes (APS). Proteins indicated in blue are causal for lysosomal storage disorders (LSDs) but are also linked to PD. The primary syndromes linked to the proteins are indicated in red. The lysosomal integral membrane protein 2 (LIMP-2) is involved in the transport of glucocerebosidase (GCase) from the endoplasmic reticulum to the lysosome. Once in the lysosome, GCase catalyzes the breakdown of glucosylceramide (GluCer) to ceramide and glucose. Ceramide is also obtained by acid sphingomyelinase (ASM) which catalyzes the hydrolysis of sphingomyelin to phosphocholine and ceramide, and GALC which hydrolyzes galactolipids, including galactosylceramide. Both α-synuclein (α-syn) and PLA2G6 have also been associated with ceramide levels, though the exact mechanisms are still unknown. Meanwhile, NPC1 has been associated with GluCer levels, even though NPC1 primarily mediates together with NPC2 intracellular cholesterol trafficking. Furthermore, ATP10B is involved in translocating GluCer towards the cytosolic membrane leaflet. Both ceramide and GluCer levels appear to play an important central role in PD pathogenesis. Meanwhile, NAG degrades heparan sulfate glycosaminoglycans by hydrolyzing terminal N-acetyl-D-glucosamine residues. Finally, VPS13C is a glycerolipid transporter between the endoplasmic reticulum and lysosomes

LSDs are Mendelian-inherited metabolic disorders caused by dysfunction in lysosomal biogenesis or function resulting in the abnormal accumulation of non-degraded substrates. More than 50 LSDs exist with a broad spectrum of clinical manifestations depending on the specific substrate and the location of substrate accumulation caused by protein deficiencies associated with lysosomal function, including proteins involved in lipid metabolism (Fig. 3). Besides α-synuclein, Lewy body pathology consists of crowded membranes and lipids originating form vesicles and fragmented organelles, including mitochondria and lysosomes [306]. Compelling associations between parkinsonism and LSDs are emerging from clinical epidemiology and genetic discoveries (Table 2). Progressive cognitive and motor decline is present in more than two-thirds of LSDs, often including parkinsonism [305].

**Table 2:** Lysosomal storage disorder genes which have been genetically linked to Parkinson disease

Gene	Phenotype	Protein product	Pathway	Reference
*ARSB*	Maroteaux-Lamy syndrome	Arysulfatase B	Mucopolysaccharide metabolism	[145, 363]
*ASAH1*	Farber lipogranulomatosis	N-acylsphingosine amidohydrolase	Sphingolipid metabolism	[173, 280]
*CTSD*	Neuronal ceroid lipofuscinoses 10	Cathepsin D	Sphingolipid metabolism	[280, 315, 327]
*GALC*	Krabbe disease	Galactosylceramidase	Sphingolipid metabolism	[50, 292]
*GBA*	Gaucher disease	Glucocerebosidase	Sphingolipid metabolism	[313, 344]
*GRN*^*a*^	Neuronal ceroid lipofuscinoses 11	Progranulin	Unknown lysosomal function	[229, 319]
*GUSB*	Mucopolysaccharidosis VII	β-glucuronidase	Mucopolysaccharide metabolism	[229, 342]
*MCOLN1*	Mucolipidosis IV	Mucolipin-1	Mucolipid metabolism	[18, 55]
*NAGLU*	Sanfilippo syndrome B	α-N-acetylglucosminidase	Mucopolysaccharide metabolism	[365, 377]
*NEU1*	Mucolipidosis I (Sialidosis)	α-neuraminidase	Mucolipid metabolism	[33, 229]
*NPC1/NPC2*	Niemann-Pick disease type C	NPC intracellular cholesterol transporter 1/2	Cholesterol trafficking	[47, 171, 232]
*SCARB2*	Action myoclonus-renal failure syndrome	Lysosomal integral membrane protein 2	Lysosomal targeting of glucosylceramidase	[24, 81]
*SLC17A5*	Salla disease, infantile sialic acid storage disorder	Sialin	Mucolipid metabolism	[280, 350]
*SMDP1*	Niemann-Pick disease type A/B	Acid sphingomyelinase	Sphingolipid metabolism	[106, 195]

^*a*^Heterozygous mutations cause autosomal dominant frontotemporal lobar degeneration with ubiquitin-positive inclusions

#### GBA

Homozygous and compound heterozygous loss-of-function mutations in *GBA* cause Gaucher disease (GD), the most common autosomal recessive LSD [344]. Clinical observations indicated parkinsonian features in a subset of GD patients and in heterozygous GBA relatives of GD patients, suggesting a role for *GBA* in the genetic etiology of PD. Indeed, a multicenter genetic analysis in 2009 confirmed an increased risk (odds ratio 5.43) of developing PD in heterozygous and homozygous *GBA* mutation carriers [313]. The missense mutations p.Asn370Ser and p.Leu444Pro are the most frequent observed pathogenic mutations in GD and PD patients, accounting for 17–31% in Ashkenazi Jewish PD patients and up to 4.5% in other PD patients [61, 234, 313, 349]. *GBA* patient carriers present an earlier age at onset and more frequent cognitive impairment compared to idiopathic PD patients [61, 234]. Widespread and abundant diffuse neocortical Lewy body pathology can be observed in brains of heterozygous *GBA* mutation carriers [234, 262]. Moreover, the activity of glucocerebosidase (GCase), the protein encoded by GBA, was found to be significantly reduced in postmortem brain tissue of PD patients with and without heterozygous *GBA* mutations, with the most profound reduction in the *substantia nigra* [111, 227]. The lysosomal enzyme GCase is involved in sphingolipid metabolism by catalyzing the breakdown of GluCer to ceramide and glucose (Fig. 3) [27, 82]. Loss of GCase and elevated GluCer levels were shown to increase α-synuclein aggregation, though the exact mechanism is unclear [165, 217]. The role of GCase in PD pathogenesis is extensively reviewed elsewhere [27, 82].

#### SMPD1

Recessive mutations in the sphingomyelin phosphodiesterase 1 (*SMPD1*) gene are responsible for neuropathic (Type A) and non-neuropathic (Type B) Niemann-Pick disease (NPD) [195, 299], while heterozygous mutations are associated with an increased risk of developing PD [106]. NPD is characterized by hepatosplenomegaly and progressive neurodegeneration, including ataxia, cognitive decline and seizures. In the Ashkenazi Jewish population, the pathogenic mutation p.Leu302Pro in *SMPD1* was found to substantially increase risk for PD (odds ratio 9.4) [106]. More studies in different populations confirmed the association between several pathogenic *SMPD1* mutations and PD [8, 55, 66, 74, 99, 105, 367]. *SMPD1* encodes acid sphingomyelinase (ASM) which catalyzes the hydrolysis of sphingomyelin to phosphocholine and ceramide in late endosomes and lysosomes (Fig. 3). Subsequently, *SMPD1* loss-of-function mutation carriers present an accumulation of sphingomyelin within lysosomes [53]. Strangely, Niemann-Pick type B patients with bi-allelic *SMPD1* loss-of-function mutations, resulting in approximately 10% residual ASM activity [351], present rarely neurological symptoms, while a single heterozygous *SMPD1* loss-of-function mutation, anticipating 50% residual ASM activity, increases the risk for PD. However, no differences in average ASM enzymatic activity in blood of sporadic PD patients compared to control individuals were observed [8, 9]. Meanwhile, an earlier age at onset in sporadic PD patients was found to be associated with reduced ASM activity levels [8]. Knockout and knockdown of *SMPD1* in HeLa and BE (2)-M17 dopaminergic cells resulted in increased α-synuclein levels [8]. The same study provided evidence that the pathogenic p.Leu302Pro mutation impair the localization of ASM to the lysosome [8]. As the association between *SMPD1* and PD is not fully understood, the functional effects of PD associated mutations need to be investigated.

#### SCARB2

Homozygous mutations in the scavenger receptor class B member 2 (*SCARB2*) gene cause action myoclonus-renal failure syndrome (AMRF), which is an autosomal recessive progressive myoclonic epilepsy, and are associated with significantly reduced GCase activity in patients [24, 68, 374]. Given the observation that homozygous *SCARB2* mutation carriers have reduced GCase activity, heterozygous *SCARB2* mutations might modify PD risk. Two common SNPs in *SCARB2*, rs6812193 and rs6825004, have been associated with PD and DLB in several genetic studies, including GWAS (odds ratio 0.84–0.91) [7, 36, 81, 136, 220, 230]. *SCARB2* encodes the lysosomal integral membrane protein 2 (LIMP-2), a mannose-6-phosphate-independent trafficking receptor which transports GCase from the ER through the Golgi apparatus and endosomes to the lysosome (Fig. 3) [374]. However, the functional variants responsible for the association between *SCARB2* and PD risk remain to be defined.

#### GALC

Recessive mutations in the galactosylceramidase (*GALC*) gene cause Krabbe disease (KD), also known as globoid cell leukodystrophy or galactosylceramide lipidosis, which is a rare, often fatal LSD resulting in progressive damage to the white matter of the peripheral and central nervous system [292]. More than 70 disease causing mutations have been identified, including missense mutations, and small deletions and insertions [362]. Recently, variants at the *GALC* locus were significantly associated with increased PD risk in a large GWAS meta-analysis [50]. The common SNP rs8005172, 12.6 kilobases proximal to the *GALC* promoter, was the most strongly associated variant, though the functional variant responsible for the association is not yet known. Interestingly, this SNP is significantly associated with *GALC* expression in multiple tissues, including the brain [19]. Moreover, brain tissue from the twitcher mouse model for KD and from patients affected with KD identified the presence of aggregated forms of α-synuclein and ubiquitin, which are both involved in Lewy bodies [318]. GALC is a lysosomal enzyme that hydrolyzes galactolipids, including galactosylceramide and galactosylsphingosine (Fig. 3). Mutations in *GALC* result in low enzymatic activity and a decreased ability to degrade galactolipids. Since galactosylceramide is an important glycosphingolipid in myelin, the pathological consequences of the GALC deficiency in KD are almost exclusively confined to the white matter of the central and peripheral nervous systems [45, 331]. The involvement of GALC in ceramide metabolism supports a role for GALC in PD risk, though further studies are needed to confirm the association with PD, and to unravel the underlying pathomechanism leading to the development of PD.

#### NPC1/NPC2

Niemann-Pick type C (NPC) disease is a rare autosomal recessive inherited disorder with a highly variable phenotype similar to Type A/B NPD and ranging from a fatal disorder within the first few months after birth to a late onset progressive disorder with predominantly neuropsychiatric symptoms of which the diagnosis is challenging. NPC is caused by mutations in the NPC intracellular cholesterol transporter 1 (*NPC1*) gene in 95% of cases or the NPC intracellular cholesterol transporter 2 (*NPC2*) gene in the remaining 5% of cases [47, 232, 288]. More than 260 mutations have been identified in *NPC1*, mostly missense mutations affecting the luminal domain of the protein [288]. Parkinsonism has been described in several NPC patients and their relatives [57, 150, 171]. Moreover, autopsy reports have described phosphorylated α-synuclein pathology in brain tissue of NPC patients [291], suggesting a possible link between NPC and PD. However, genetic studies investigating the association between *NPC1*/*NPC2* variants and PD have shown conflicting results [171, 373].

*NPC1* encodes a large protein that resides in the late endosomes and lysosomes which mediates cholesterol efflux [185]. When cholesterol is released from low-density lipoproteins in the lumen of the late endosomes/lysosomes, it is transferred by NPC2 to the N-terminal cholesterol-binding pocket of NPC1 [141, 359]. Loss-of-function of NPC1 leads to accumulation of cholesterol in the late endosome/lysosome [155, 375].

#### NAGLU

Recessive mutations in *NAGLU* are responsible for Sanfilippo syndrome B, also known as mucopolysaccharidosis III disease B (MPS-IIIB) [377]. Patients with MPS-IIIB present early-onset progressive neurological disturbances, including mental retardation, hyperactivity and seizures, in addition to mild somatic manifestations. Interestingly, immunoreactivity for phosphorylated α-synuclein was observed in brain tissue of MPS-IIIB patients [129]. The SNP rs2071046 tagging a common haplotype of *NAGLU* was shown to be associated with an increased risk for PD (odds ratio 1.23) [365]. Moreover, in a recent large meta-analysis of GWAS, *NAGLU* was nominated as a gene of interest in novel loci significantly associated with increased risk for PD [229].

NAGLU encodes α-N-acetylglucosminidase (NAG), a lysosomal hydrolase which degrades heparan sulfate glycosaminoglycans by hydrolyzing terminal N-acetyl-D-glucosamine residues (Fig. 3). Interestingly, heparan sulfate stimulates the formation of α-synuclein fibrils in vitro [56], and in neuroblastoma cells, cellular internalization of α-synuclein amyloid fibrils is dependent on heparan sulfate [138]. Glycosaminoglycans modulate the lysosome degradation pathway by regulating CTSD, the major lysosomal protease responsible of α-synuclein degradation. In a neuroblastoma cell model of PD, elevated glycosaminoglycans levels resulted in reduced CTSD activity and intracellular accumulation of α-synuclein [190].

#### Other lysosomal storage disorder genes genetically linked to PD

The LSD genes *MCOLN1*, *ARSB*, *GUSB*, *GRN* and *NEU1* have been genetically connected with PD as well via gene based association studies, whole exome sequencing in unrelated PD patients or meta-analysis of GWAS, but await further replication [50, 145, 229]. Interestingly, a significant burden of rare LSD gene variants in PD patients was observed in a large whole exome sequencing dataset including > 1100 PD patients and > 1600 control individuals, taken into account 54 different LSD genes [280]. In this study, *SLC17A5*, *CTSD*, *ASAH1* were identified as novel candidate susceptibility genes for PD risk [280].

### Disrupted lipid homeostasis in PD pathogenesis

In recent years, lipid homeostasis has become a principal suspect in PD pathogenesis [368]. Binding of α-synuclein to membranes depends on its lipid composition, which has led to the hypothesis that PD pathology is induced by alterations in the binding properties of α-synuclein to membranes or lipid rafts. Indeed, α-synuclein is known to selectively interact phospholipids and sphingolipids, and disruption of lipid metabolism has been found to predispose α-synuclein toxicity [70, 77, 92, 93, 182, 214, 215, 276, 318, 329, 332, 333, 380].

Interestingly, both GCase and ATP10B play essential roles in the fate of lysosomal GluCer, and loss-of-function of both proteins results in the intra-lysosomal accumulation of GluCer combined with lysosomal dysfunction (Fig. 3) [97, 165, 213]. ATP10B may therefore synergize with GCase to maintain low levels of GluCer or GluSph in the lysosome. Accordingly, loss of ATP10B functionality might result in the same affected pathways associated with loss of GCase functionality. Besides lysosomal dysfunction, loss of GCase activity and elevated GluCer levels cause α-synuclein aggregation, mitochondrial impairment, inflammation, and ER stress [82, 165, 217, 314, 339], implying these pathways to be affected as a result of ATP10B dysfunctionality. Moreover, the LSD genes *SMPD1*, *GALC* and *SCARB2*, which have been associated with PD, are also involved in or linked to ceramide homeostasis (Fig. 3) [7, 50, 106]. Elevated levels of sphingolipids including GluCer, glucosylsphingosine, and sphingosine have been detected as well in NPC1 mutant cells, though the mechanism for accumulation of these lipids is still unclear (Fig. 3) [203, 340]. The brain is the organ with the highest proportion of lipid content, both in neurons and glial cells, in which ceramides are involved in a wide variety of functions to coordinate brain homeostasis, extensively reviewed elsewhere [62, 265]. Ceramide is enriched in lipid rafts, which are specialized membrane micro-domains, acting as assembling hubs for signaling complexes, to enable compartmentalization of various cellular processes [303]. Accumulation of ceramide in neuronal rafts was shown to be associated with impaired receptor trafficking and synapse loss [134].

Additionally, cholesterol homeostasis has been associated with PD pathogenesis, through the genetic association between *NPC1* and PD (Fig. 3). Loss of NPC1 function has been shown to impede the clearance of α-synuclein [89, 172, 198], and accumulated oxidized metabolites of cholesterol, which have been identified in Lewy body brain pathology, can directly induce α-synuclein fibrilization [34]. Accumulated lysosomal cholesterol levels have been reported in fibroblasts of PD patient carriers with the *GBA* p.Asn370Ser pathogenic mutation [109]. Vice versa, increased intracellular cholesterol has been shown to initiate the breakdown of GCase via ER-associated degradation in proteasomes, which in turn resulted in reduced lysosomal GCase levels and increased glucosylceramide and α-synuclein levels [282].

Lysosomal PC export is also diminished by ATP10B dysfunction [213], which is in line with the observation of a disturbed PC lipid homeostasis in the *substantia nigra* of a PD rat model [94]. Loss of function mutations in PLA2G6, which is involved in oxidative damage repair to membrane phosphopholipids, membrane remodeling and iron homeostasis, also disturb neuronal lipid homeostasis, including ceramide levels (Fig. 3) [17, 200, 252, 309].

Finally, VPS13C enables the transport of glycerolipids between the ER and late endosomes/lysosomes, implicating defects in membrane lipid homeostasis and lysosomal dysfunction as a consequence of loss of VPS13C functionality (Fig. 3) [183].

### Crosstalk between vesicular transport, lysosomes and mitochondria in Parkinson disease

Causal PD mutations in genes encoding proteins involved in one PD associated pathway are frequently associated secondary effects in other PD related pathways. The autophagy-lysosomal pathway is the only means by which damaged mitochondria are turned [15] and therefore, disruption in autophagy or lysosomal dysfunction could result in, if not exacerbate, mitochondrial dysfunction. Conversely, lysosomal function could be influenced by mitochondrial quality control, dynamics and/or respiration. Indeed, emerging evidence indicate that autophagy-lysosomal dysfunction impairs mitochondrial homeostasis, and in turn, mitochondrial defects also impact lysosomal functions, suggesting a complex relationship between these processes [260].

#### Mitochondrial dysfunction caused by alteration in genes primarily involved in transport pathways and lysosomal functioning

Increased levels of wild-type α-synuclein and α-synuclein with PD causing mutations have been associated with mitochondrial fragmentation and ROS accumulation. Moreover, α-synuclein has been found to localize at mitochondria-associated membranes (MAM), junctions that physically connect ER with mitochondria that are involved in Ca^2+^ signaling and apoptosis [122]. Pathogenic mutations in α-synuclein reduce the localization to MAM, and increase mitochondrial fragmentation, suggesting a direct role for α-synuclein in mitochondrial morphology [122]. Indeed, overexpressing wild-type or mutant α-synuclein was found to dissociate the ER and mitochondria at MAM, thereby impairing Ca^2+^ exchange and mitochondrial energy production [248]. Additionally, α-synuclein might regulate the PGC1α transcriptional network, involved in mitochondrial biogenesis and apoptosis. In a patient-derived stem cell model of PD carrying the *SNCA* p.Ala53Thr mutation, basal and mitochondrial toxin-induced nitrosative/oxidative stress resulted in S-nitrosylation of transcription factor myocyte-specific enhancer factor 2C (MEF2C), thereby inhibiting the transcription of PGC1α [289].

LRRK2 interacts with a number of key regulators of mitochondrial dynamics of fission and fusion, in the cytosol or at mitochondrial membranes. In mouse cortical neurons and human neuroblastoma cells, endogeneous LRRK2 interacts with the mitochondrial fission factor Dynamin like protein 1 (DLP1). DLP1 phosphorylates and transfers from the cytosol to mitochondria upon LRRK2 expression, resulting in mitochondrial fission. LRRK2 also interacts with the mitochondrial fusion regulators mitofusin 1 and mitofusin 2, and OPA1, a mitochondrial Dynamin like GTPase. Even though one study reported a direct interaction between LRRK2 and parkin in HEK293T cells [320], this finding could not be confirmed elsewhere [65]. However, LRRK2 is connected to the PINK1/parkin mediated mitophagy pathway via its substrate Rab10. Indeed, it was recently demonstrated that Rab10 binds the autophagy receptor optineurin (OPTN), promotes OPTN accumulation on depolarized mitochondria and thereby facilitating autophagosome formation around mitochondria, and consequently mitophagy [360]. Expression of LRRK2 p.Gly2019Ser in mouse cortical neurons causes defects in mitochondrial morphology and dynamics [237, 324]. Moreover, postmortem brain tissue of PD patients carrying the p.Gly2019Ser mutation demonstrates decreased levels of mature OPA1 [324]. In fibroblasts of PD patients with either the *LRRK2* p.Gly2019Ser or p.Arg1441Cys mutation mitophagy of depolarized mitochondria is impaired [360].

*VPS35* deficient mouse dopaminergic neurons and human fibroblasts also results in defects in mitochondrial fusion and mitochondrial fragmentation [338, 356]. VPS35-induced mitochondrial deficits can be prevented by inhibition of mitochondrial fission [356]. Moreover, VPS35 mutants show an increased interaction with the mitochondrial fission factor DLP1 [356].

*VPS13C* knockdown in COS-7 and in HEK293T cells resulted in mitochondrial fragmentation, decreased mitochondrial membrane potential, increased respiration rates and exacerbated PINK1/parkin-dependent mitophagy [193].

Knockdown of *ATP13A2* in primary mouse cortical neurons and in SH-SY5Y cells shows an increase in mitochondrial fragmentation and an increase production of ROS [128, 278]. These effects could be mimicked by inhibiting autophagy induction using siRNA to Autophagy Related 7 (ATG7), a protein required for autophagy [128]. These results demonstrate that a decrease in autophagy influences mitochondrial quality control pathways, resulting in increased ROS production. Oppositely, overexpression of wild-type *ATP13A2* in cultured midbrain dopaminergic neurons delays cadmium-induced mitochondrial fragmentation in neurons, consistent with a neuroprotective effect [278].

Knocking out *PLA2G6* in mice results in degeneration of mitochondrial inner membranes and presynaptic membranes, triggering mitochondrial and synaptic dysfunction, and significant iron accumulation in brain [20–22].

Neuronal and glial cells of conditional *GBA* knockout mice present mitochondrial fragmentation, reduced respiratory chain complex activities, decreased mitochondrial membrane potential and lower oxygen consumption [244]. Analogous, overexpressing *GBA* p.Leu444Pro in SHSY-5Y neuroblastoma cells and knock-in of heterozygous *GBA* p.Leu444Pro in mice both trigger mitochondrial dysfunction by inhibiting autophagy and mitochondrial priming, a process by which autophagy receptor proteins are recruited to damaged mitochondria for degradation [196]. iPSC-derived dopaminergic neurons from GD and PD patients with GBA mutations showed increased glucosylceramide and α-synuclein levels, autophagic and lysosomal defects and a dysregulation of Ca^2+^ homeostasis [296]. Mitochondrial activities are driven in a Ca^2+^ dependent manner, and alterations in Ca^2+^ homeostasis may imply mitochondrial dysfunction [116, 286]. Induced mitophagy with carbonyl cyanide-m-chlorophenyl-hydrazine (CCCP) in 3D-neurosphere-models, consisting of neural stem cells with heterozygous and homozygous GBA p.Asn370Ser, resulted in a significant increase in lysosomal transcription factor EB (TFEB) mRNA levels, the master regulator of lysosomal and autophagy genes [224]. Interestingly, PGC1α mRNA levels were also significantly increased following CCCP-treatment in heterozygote, but not homozygote neurospheres, which might be explained by compensatory mechanism absent in homozygous lines [224]. In mouse cortical neurons, the chaperone ambroxol, which increases GCase mRNA levels and lysosomal GCase activity, was also shown to increase TFEB and PGC1α levels, block macro-autophagy flux and increased exocytosis [209]. These findings suggest that the GCase chaperone ambroxol might act on different pathways, including mitochondrial and lysosomal biogenesis, and the secretory pathway. Of note, most LSDs associated with PD or parkinsonism present some degree of mitochondrial dysfunction associated with primary lysosomal impairment [266].

Recently, proteome analysis of primary rat cortical neurons either overexpressing or silencing the lysosomal receptor LAMP2A, resulting in alterations in chaperone-mediated autophagy, identified a more than 2-fold difference in DJ-1 expression compared to control conditions [38]. Moreover, LAMP2A silencing, which results in DJ-1 depletion, sensitizes neurons to oxidative stress [38].

#### Endo-lysosomal dysfunction caused by alteration in genes primarily involved in mitophagy, mitochondrial dynamics and oxidative stress control

A mouse model of mitochondrial dysfunction, generated by deleting the mitochondrial transcription factor A (TFAM) in CD4^+^ T cells, demonstrated that mitochondrial respiratory defects impair lysosomal function, endo-lysosomal trafficking and autophagy, and increase sphingomyelin levels [16]. Similarly, large lysosomal vacuoles and lysosomal dysfunction were observed in brains from mice lacking the mitochondrial protein apoptosis inducing factor (AIF) and in embryonic fibroblasts from *OPA1* knockout mice [73]. The lysosomal defects in the mouse embryonic fibroblasts were partially rescued by treatment with the antioxidants N-acetylcysteine or coenzyme Q10, suggesting that increased ROS from damaged mitochondria mediates lysosomal dysfunction [73]. Treatment of the mouse motor neuron NSC-34 cell line with the mitochondrial complex I inhibitor rotenone causes alterations in lysosomal biogenesis, function and morphology [123]. Interestingly, inducing TFEB via trehalose treatment in iPSC-derived dopaminergic neurons with compromised mitochondrial functioning, caused by long-term treatment with rotenone, restored the mitochondrial membrane potential and ATP production [312].

While parkin is well known to be involved in the regulation of mitophagy and mitochondrial biogenesis, the protective activity of parkin has been broadened to include roles in lipid metabolism and fat uptake [147, 164]. Moreover, parkin regulates the endo-lysosomal pathway by ubiquitinating the late-endosomal GTPase Rab7, which is a regulator of lysosomal dynamics [137, 321]. Loss of parkin function in primary fibroblasts of two PD patients with homozygous *PARK2* mutations caused decreased endosomal tubulation and endosomal membrane association of VPS35 and sorting nexin 1, suggesting impairment of retromer pathway [321]. *Substantia nigra* tissue of *PARK2* p.Q311* mutant mice displayed a late-stage block in autophagy, an increased PARIS expression and a PARIS-dependent reduced expression of both PGC1α and the lysosomal transcription factor TFEB [312]. Moreover, primary fibroblasts of a patient with juvenile PD with compound heterozygous deletions in *PARK2* displayed abnormal abundance, acidification and morphology of the late endocytic compartment and lysosomal dysfunction [123].

PINK1 depletion in mouse embryonic fibroblasts from *PINK1* knockout mice also impaired lysosomal activity and led to enlargement of lysosomal vacuoles [73] and silencing of *DJ-1* in M17 neuroblastoma cells led to an accumulation of autophagy markers, in addition to mitochondrial membrane depolarization and mitochondrial fragmentation [341]. Moreover, a recent study in dopaminergic neurons derived from PD patients with homozygous *DJ-1* p.E64D identified a time dependent pathological cascade starting with mitochondrial oxidative stress, followed by oxidized dopamine accumulation and finally resulting in reduced GCase activity, lysosomal dysfunction and α-synuclein accumulation [41].

#### Synergistic connection between the lysosomal and the mitochondrial compartment

Altogether, these data suggest synergistic effects of mitochondrial and lysosomal dysfunction in the pathogenesis of PD. The crosstalk between both pathways was also observed in a miRNA expression analysis to investigate the role of miRNAs in PD pathogenesis. In 6-hydroxydopamine induced stress conditions in SH-SY5Y cells, miR-5701 was shown to be significantly downregulated, and putative targets of miR-5701 are genes involved in lysosomal biogenesis and mitochondrial quality control [271]. The transfection of miR-5701 in SH-SY5Y cells induced both mitochondrial dysfunction and defects in autophagy flux [271]. The observed decrease in miR-5701 in 6-hydroxydopamine induced stress conditions might be a compensatory mechanism to simultaneously rescue lysosomal and mitochondrial function. Lastly, dynamic contact sites between lysosomes and mitochondria were recently identified using live-cell imaging, which was promoted by active GTP-bound lysosomal Rab7, a regulator of lysosomal dynamics [137, 366]. Moreover, these mitochondrial-lysosomal contact sites marked sites of mitochondrial fission, suggesting Rab7 may regulate mitochondrial dynamics [366]. Mitochondrial-lysosomal contact sites may be involved in a bidirectional regulation of mitochondrial and lysosomal dynamics, and might partially explain dysfunction of both organelles in PD.

### Oligogenic and polygenic involvement of PD and APS genes

Because genes involved in mitochondrial and lysosomal function are associated with PD, and because of substantial evidence highlighting the crosstalk between these two pathways, it is reasonable to consider that oligogenic or polygenic inheritance of genes implicated in the mitochondrial-lysosomal pathway contribute to the genetic etiology of PD. Indeed, accumulating clinical and genetic observations suggest that besides monogenic inheritance, caused by dominant or recessive mutations in a single gene, more complex inheritance patters of familial PD exist.

#### Evidence pointing towards more complex inheritance patterns for PD

In 12 non-consanguine families affected with PD from Crete in which PD originated from both parental sides a high proportion (43%) of bilineal siblings were affected with PD, whereas only 5.7% of their offspring were affected [322]. This observation suggest recessive oligogenic inheritance in which disease predisposing alleles from two or more genes need to be present in the same individual for the expression of PD. Such alleles will be diluted again in the succeeding unilineal generation, resulting in a reduced proportion affected offspring. Moreover, single heterozygous mutations in *PARK2*, *PINK1*, *DJ-1* and *ATP13A2* are significantly more prevalent in PD or EOPD patients compared to control individuals [4, 75, 80, 133, 154, 170, 199, 238, 322, 330], suggesting these variants increase risk for PD, act as onset modifiers for PD, or contribute to the disease together with other mutations in an oligogenic fashion.

#### Oligogenic mutations in PD

In two Japanese families affected with autosomal recessive PD caused by homozygous or compound heterozygous *PARK2* mutations, a heterozygous *PINK1* mutation was identified as well [100]. The age at onset of patients with digenic mutations was lower than the age at onset of patients with the same recessive *PARK2* mutations alone, indicating that heterozygous *PINK1* might act as a modifier of age at onset in *PARK2* mutation carriers. This study identified as well a sporadic PD patient with single heterozygous mutations in *PARK2* and *PINK1* [100]. Digenic heterozygous *PARK2*-*PINK1* mutations have also been reported in two affected siblings with EOPD and one unrelated EOPD patient from Mexico [223]. These causative digenic *PARK2*-*PINK1* mutations could be explained by the parkin/PINK1 pathway in which PINK1 acts directly upstream of parkin in regulating mitochondrial quality control and dynamics (Fig. 2). Similarly, digenic heterozygous *PINK1* and *DJ-1* missense mutations have been reported in a recessive family with two affected siblings [337]. This study also showed that PINK1 and DJ-1 physically associate and cooperate to protect cells against oxidative stress [337].

Most frequently reported to date are digenic *PARK2*-*LRRK2* mutation carriers. So far, two carriers of *LRRK2* p.Gly2019Ser and homozygous *PARK2* mutations have been reported: a carrier of a homozygous deletion of exon 4–5-6 and a carrier of a homozygous triplication of exon 2 in *PARK2* [108, 194]. The clinical manifestations of both patients are as typically seen in other *PARK2* mutation carriers rather than in other *LRRK2* mutation carriers, including dopa-responsive parkinsonism and an early age at onset. Additionally, several cases of digenic heterozygous *LRRK2* and *PARK2* mutations have been reported [65, 194, 238]. However, digenic *PARK2*-*LRRK2* heterozygous mutations do not seem to cause a more severe disease progression or an earlier age at onset compared to single heterozygous *PARK2* or *LRRK2* mutations [65, 194, 238]. Noteworthy, one at risk member aged 52 of a family affected by the *LRRK2* p.Gly2019Ser mutation carried as well as a heterozygous frameshift mutation in *PARK2* and the pathogenic *GBA* p.Asn370Ser mutation [250]. The *PARK2* and *GBA* mutations were absent from other members of the family and were likely inherited from the parent married into the family [250].

While the connection between PINK1 and SYNJ1 is yet unclear, digenic heterozygous mutations *SYNJ1* p.Ser1422Arg and *PINK1* p.W437* have been identified in a Brazilian EOPD patient [273].

The impact of oligogenic mutations on disease expression is currently not well understood due to the limited number oligogenic mutation carriers identified so far and the lack of large families to investigate segregation with PD. Disease expression in oligogenic mutation carriers is probably a joint effect of genetic background, gene-gene and gene-environment interactions.

#### Rare variant analysis suggests oligogenic and polygenic inheritance of PD

Rare variant analysis in 7900 PD patients with and without a known pathogenic mutation and 6166 control individuals revealed that more than 30% of PD patients with a recognized primary genetic cause of the disease had additional rare variants in Mendelian PD genes [207]. The carriers of additional rare variants in the PD genes had younger ages at onset of approximately 4 to 6 years, though this was not significant (*p* > 0.05) [207]. Exome sequencing analysis of postmortem human brains of 58 DLB and 39 PD patients identified a significant enrichment of rare oligogenic variants in neurodegenerative brain diseases (PD, DLB, Alzheimer’s disease, frontotemporal dementia and amyotrophic lateral sclerosis) genes in PD/DLB patients (23.71%) compared to control individuals (10.22%) [159]. Moreover, significant polygenic enrichment in PD patients of rare, non-synonymous variants of a gene-set of proteins involved in mitochondrial DNA maintenance was identified in both a Norwegian and a North American cohort consisting of PD patients and control individuals [104]. All of the above-mentioned observations suggest oligogenic and polygenic inheritance contribute to the expression of PD and might explain a part of the missing heritability in PD. Moreover, these observations may provide new insights for functional research to investigate how PD pathways are interconnected.

## Concluding remarks

Numerous genes, either causing PD, APS or increasing risk, are directly and indirectly associated with defects in vesicular transport pathways, lysosomal dysfunction and mitochondrial dysfunction. Additionally, increasing evidence suggests a link between numerous LSDs and PD, though further clinical, genetic and biochemical studies are needed to clarify these associations. Nevertheless, these emerging associations highlight the involvement of lipid homeostasis and especially ceramide homeostasis in PD pathogenesis. However, why homozygous mutations in LSD genes cause LSDs, whether or not associated with neurological symptoms, and heterozygous mutation increase PD risk is unclear. We hypothesize that genetic modifying factors may influence the phenotype associated with these LSD gene mutations.

Our current understanding is that the lysosomal and the mitochondrial compartment is highly interconnected, given that a primary defect in either compartment leads to dysfunction of the other compartment. Moreover, while mitochondria-ER contact sites have been recognized for years [86], and recently implicated in PD pathogenesis via the discovery of VPS13C as a lipid transport tether between these two organelles [183], mitochondrial-lysosomal contact sites are only recognized more recently. However, dynamic contact sites and crosstalk may be important to transport metabolites and ions between the two organelles, including lipids, as seen for VPS13C [183]. Moreover, transcriptional regulation seems to play an important role in the crosstalk between the mitochondria and lysosomes, indicated by alterations in protein levels of the lysosomal and mitochondrial transcription factors, TFEB and PGC1α respectively, as a result of PD/APS gene defects and impairment of these organelles. Despite new evidence, significant gaps remain in our understanding of the functional and physical associations between these PD associated pathways [366].

Oligogenic mutations of the mitochondrial-lysosomal pathway have been identified in PD patients but have not been extensively studied yet. However, current observations suggest more complex inheritance patters of genes of interconnected PD pathways, further strengthening the crosstalk and synergistic connection between these pathways in PD pathogenesis. Nevertheless, segregation analysis, clinical and biochemical studies are needed provide insights in gene-gene interactions leading to the development of PD. The genetic architecture of PD, in terms of the number of variants needed to reach the threshold to express the disease and their respective effects size, probably ranges from monogenic highly penetrant variants, perhaps influenced by modifiers, to oligogenic rare variants with high to moderate effect sizes, to polygenic common risk factors which could also act as modifiers of more penetrant variants. Therefore, the presence of a mutation in one gene should not be an exclusion criterion for further genetic screening in PD patients. Mainly in PD patients with a single heterozygous mutation in a recessive gene, additional screening could reveal oligogenic variants in the same or connecting pathways. Possible oligogenic inheritance patterns may include both PD/APS genes and LSD genes associated with an increased risk for the development of PD.

## Data Availability

Data sharing is not applicable to this article as no datasets were generated.

## Abbreviations

AIF: Apoptosis inducing factor
AMRF: Action myoclonus-renal failure syndrome
APS: Atypical parkinsonian syndromes
ASM: Acid sphingomyelinase
ATG7: Autophagy Related 7
ATP10B: ATPase class V type 10B
CCCP: Cyanide-m-chlorophenyl-hydrazine
CDC50A: Cell Cycle Control Protein 50A
CI-MPR: Cation-independent mannose 6-phosphate receptor
CRC: Cargo-recognition complex
CTSD: Cathepsin D
DLB: Dementia with Lewy bodies
DLP1: Dynamin like protein 1
EOPD: Early-onset Parkinson’s disease
ER: Endoplasmic reticulum
FBXO7: F-box protein 7
GALC: Galactosylceramidase
GCase: Glucocerebosidase
GD: Gaucher disease
GluCer: Glucosylceramide
GWAS: Genome-wide association studies
INAD: Infantile neuroaxonal dystrophy
iPLA-β: Calcium-independent group VI phospholipase A2
iPSC: Induced pluripotent stem cell
KD: Krabbe disease
LIMP-2: Lysosomal integral membrane protein 2
LSD: Lysosomal storage disorders
MAM: Mitochondria-associated membrane
MEF2C: Myocyte-specific enhancer factor 2C
MPS-IIIB: Mucopolysaccharidosis III disease B
NAD: Neuroaxonal dystrophy
NAG: α-N-acetylglucosminidase
NPD: Niemann-Pick disease
NPC1: Niemann-Pick type C intracellular cholesterol transporter 1
NPC2: Niemann-Pick type C intracellular cholesterol transporter 2
OPTN: Optineurin
PARIS: Parkin interacting substrate
PARL: Presenilin-associated rhomboid-like protein
PC: Phosphatidylcholine
PD: Parkinson’s disease
PGC1α: Proliferator-activated receptor gamma coactivator 1-α
PINK1: PTEN-induced putative kinase 1
PIP_2_: Phosphatidylinositol 4,5-bisphosphate
PIPs: Phosphoinositide phosphates
PLA2G6: Phospholipase A2 group 6
PTC: Premature termination codon
ROS: Reactive oxygen species
SCARB2: Scavenger receptor class B member 2
SCF: SKP1-cullin-F-box
SMPD1: Sphingomyelin phosphodiesterase 1
SNPs: Single nucleotide polymorphisms
TFAM: Mitochondrial transcription factor A
TFEB: Transcription factor EB
UbR: Ubiquitin-related
VPS13C: Vacuolar protein sorting 13 C
